# Wide Band Gap Polymer Based on Indacenodithiophene and Acenaphthoquinoxaline for Efficient Polymer Solar Cells Application

**DOI:** 10.3390/polym9110578

**Published:** 2017-11-07

**Authors:** Ming Liu, Zhitian Liu, Yong Zhang, Liancheng Zhao

**Affiliations:** 1 School of Materials Science and Engineering, Wuhan Institute of Technology, Wuhan 430205, China; mingliu016@163.com; 2 School of Materials Science and Engineering, Harbin Institute of Technology, Harbin 150001, China; lczhao@hit.edu.cn

**Keywords:** indacenodithiophene, acenaphthoquinoxaline, conjugated polymer, polymer solar cells

## Abstract

A new wide band gap polymer PIDT-AQx with indacenodithiophene (IDT) as the electron-rich unit and acenaphthoquinoxaline (AQx) as the electron-deficient unit has been designed and synthesized. The optical band gap of PIDT-AQx was 1.81 eV with a HOMO energy level of −5.13 eV. Polymer solar cells with the blend of PIDT-AQx/PC_71_BM as the active layer achieved a power conversion efficiency (PCE) of 4.56%, with an open-circuit voltage (*V*_oc_) of 0.84 V, a current density (*J*_sc_) of 9.88 mA cm^−2^, and a fill factor (FF) of 55% without any solvent additives and pre- or post-treatments. The photovoltaic performance of PIDT-AQx could be slightly improved with a PCE up to 4.78% after thermal annealing due to enhanced *J_sc_*. The results indicate that acenaphthoquinoxaline is a promising building block for developing conjugated polymers for efficient solar cells application.

## 1. Introduction

Conjugated polymer solar cells (PSCs), with advantages such as low cost, light weight, flexibility, and roll-to-roll printing technology, have been intensely investigated as one of the potential renewable energy sources in recent years [1,2,3,4,5,6,7,8,9,10,11,12]. At present, the most typical and successful polymer solar cell is the so-called bulk heterojunction (BHJ) photovoltaic cell, of which the active layer is based on a blend film of a conjugated polymer donor and a fullerene derivative (or non-fullerene) acceptor [13,14,15,16]. To improve the photovoltaic performance of BHJ PSCs, considerable researches on designing a new polymer donor (or non-fullerene acceptor) and optimizing the device-processing technology have been done [17,18,19,20,21,22]. One of the most successful ways to design a conjugated polymer with good photovoltaic performance is the donor–acceptor (D–A) approach, where the polymer backbone consists of an electron-rich donor unit and an electron-deficient acceptor unit alternatively [23,24,25,26,27,28]. Meanwhile, the morphological optimization of the active layer in PSCs also plays a key role to improve the photovoltaic performance by facilitating the charge generation, separation, and transport within the device [29,30,31,32]. Various methods such as thermal annealing, solvent annealing, and adding additives have been used to control the morphology to achieve favorable phase separation. However, these methods will raise the difficulty in device engineering and are negative for large-area production. Therefore, it will be much more important if a promising device performance could be achieved from the pristine blend film. 

Benefiting from the development of numerous D–A conjugated polymers, the power conversion efficiencies (PCEs) of the BHJ PSCs have now increased up to ~13% after the simultaneous optimizations on materials and device engineering [33,34]. Up to now, a great deal of conjugated polymers have been designed and synthesized based on abundant building blocks, including both the electron-rich and electron-deficient moieties. Indacenodithiophene (IDT), with high coplanarity and an extended conjugation structure, has been widely applied in the synthesis of high-performance *p*-type polymers [35,36]. The PCEs of IDT-based polymers have broken 8% in single-junction BHJ devices and a more encouraging PCE of >11% in tandem solar cells [37]. Acenaphtho[1,2-b]quinoxaline (AQx) is an extended derivative of the quinoxaline unit with a strong electron withdrawing property from the two imine nitrogen atoms, and could also form the relatively stable quinoidial structure. However, the AQx unit is seldom used to construct D–A conjugated polymers for PSCs. Currently, only a few AQx-based polymers have been reported, and they possessed inferior photovoltaic performance, with a PCE of less than 5% [38,39,40]. For example, Huang et al. reported benzodithiophene (BDT) and acenaphthoquinoxaline-based polymer, which only achieved a PCE of 1.2% [38]. Recently, Bo et al. synthesized two poly(acenaphthoquinoxaline-*co*-benzodithiophene) polymers, and the polymer solar cells based on the blend of the polymers and PC_71_BM as the active layer showed PCE of 1.65%–4.72% [39].

**Figure 1:**
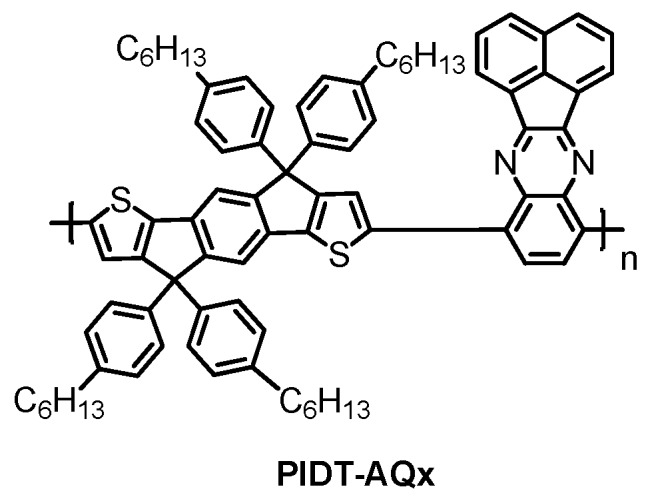
The chemical structure of PIDT-AQx in this work.

In this work, we copolymerized the IDT and AQx units to afford a new wide band gap alternative conjugated polymer PIDT-AQx (Figure 1). The polymer has a stronger absorption range, from 300 nm to 700 nm, and the optical band gap was measured to be 1.81 eV according to the absorption edge. The HOMO and LUMO energy levels of PIDT-AQx were measured to be −5.13 eV and −3.25 eV, respectively. PSC devices were fabricated based on the PIDT-AQx/PC_71_BM blend. It was found that when the active layer was processed directly from the solution without any pre- or post-treatment or additional solvent additives, a power conversion efficiency (PCE) of 4.56% was obtained with an open-circuit voltage (*V*_oc_) of 0.84 V, a current density (*J*_sc_) of 9.88 mA cm^−2^, and a fill factor (FF) of 55% for the pristine device. The results indicate that the acenaphthoquinoxaline unit could be a promising building block in pursing high-performance conjugated polymer solar cells with a simple processing technique. In addition, the photovoltaic performance was slightly improved to 4.78% with a higher *J_sc_* after thermal annealing at 110 °C for 10 min, which is, to the best of our knowledge, among the best PCEs reported for AQx-based PSCs.

## 2. Experimental Section

### 2.1. Materials and General Characterization Methods

Monomers 3 (AQx) [41] and 5 (IDT-Sn) [42] were synthesized according to the literature methods. All other chemicals were commercially available and used without further purification, unless otherwise indicated. The ^1^H-NMR spectra were collected on a Bruker AV400 NMR spectrometer (Bruker, Karlsruhe, Germany) in CDCl_3_ solution with tetramethylsilane (TMS) as an internal standard. The UV-Vis absorption spectra of the polymer was obtained using a TU-1901 spectrophotometer (Persee, Beijing, China). Cyclic voltammetry (CV) of the polymer film was conducted in 0.1 M tetrabutylammonium hexafluorophosphate in acetonitrile, at a scan rate of 100 mV/s with ITO, Pt wire, Ag/AgCl, as the working electrode, counter electrode, and reference electrode, respectively. The molecular weight and distribution were determined by Gel Permeation Chromatography (GPC), with tetrahydrofuran (THF) as the eluent and monodispersed polystyrene as the standard. Atomic force microscopy (AFM) images under tapping mode were recorded from the multimode scanning probe microscope (AFM5100N, HITACHI, Tokyo, Japan).

### 2.2. Device Fabrication and Characterization

Conventional solar cells based on PIDT-AQx/PC_71_BM were fabricated with a device architecture of ITO/PEDOT:PSS/PIDT-AQx:PC_71_BM/Ca/Al. Under ultrasonic conditions, the ITO-coated glass substrates (15 Ω/sq.) were cleaned with detergent, de-ionized water, acetone, and isopropyl alcohol each for 15 min in sequence. A thin layer (ca. 40 nm) of PEDOT:PSS (Baytron^®^ P VP AI 4083 (H. C. Starck, GmbSH, Leverkusen, Germany), filtered through a 0.45 μm poly(tetrafluoroethylene) filter) was then spin-coated on the pre-cleaned ITO-coated glass substrates at 5000 rpm and baked at 140 °C for 10 min under ambient conditions. In an argon-filled glove-box, the PIDT-AQx:PC_71_BM active layer (ca. 90 nm) was spin-coated on the PEDOT:PSS layer at 900 rpm from a homogeneously blended solution, which was prepared by dissolving the polymer and PC_71_BM at a weight ratio of 1:3 in *o*-dichlorobenzene. Before electrode deposition, the substrates were annealed at 110 °C for 10 min. Then, the substrates were pumped down to high vacuum (<2 × 10^−6^ Torr), and calcium (30 nm) topped with aluminum (100 nm) was thermally evaporated onto the active layer through shadow masks to define the active area of the devices. The current density–voltage curves were measured on with a Keithley 2420 source meter (Keithly, Cleveland, OH, USA) under simulated AM 1.5 G solar irradiation at 100 mW/cm^2^ (Class AAA solar simulator, 94023A-U, Newport, Irvine, CA, USA) and the external quantum efficiency (EQE) was measured by an Oriel Newport System. 

### 2.3. Synthesis of PIDT-AQx

Synthesis of PIDT-AQx. In a 25 mL flask, 5 (IDT-Sn, 250 mg, 0.2 mmol), 3 (AQx, 84 mg, 0.2 mmol), Pd_2_(dba)_3_ (4 mg, 0.004 mmol), and P(*o*-tol)_3_ (8 mg, 0.026 mmol) were added. The flask was then degassed with nitrogen for 20 min, and anhydrous toluene (6 mL) was added using a syringe under nitrogen. The reaction mixture was then degassed with nitrogen for another two times, and heated to 110 °C for 48 h. For end-capping, 2-(tributylstannyl)thiophene (0.2 mL) was added into the mixture and stirred for 12 h; 2-bromothiophene (0.5 mL) was then added and stirred for another 12 h. After cooling to room temperature, the mixture was poured into methanol. The precipitation was collected, dissolved into toluene, and then passed through a short kieselguhr column. The toluene solution was concentrated and poured into acetone. The precipitation was collected, washed with acetone, and dissolved into toluene again. The toluene solution was concentrated and poured into petroleum ether. The collected solid was then dried over vacuum to give PIDT-AQx (172 mg, 73%). The ^1^H-NMR (400 MHz, CDCl_3_): δ (ppm) = 8.52–8.44 (m, 2H), 8.1 7−8.05 (m, 4H), 7.91–7.84 (m, 4H), 7.68–7.30 (m, 10H), 7.18–7.08 (m, 8H), 2.61 (m, 8H), 1.63–1.28 (m, 32H), 0.85 (m, 12H). 

## 3. Results and Discussion

### 3.1. Synthesis

**Scheme 1:**
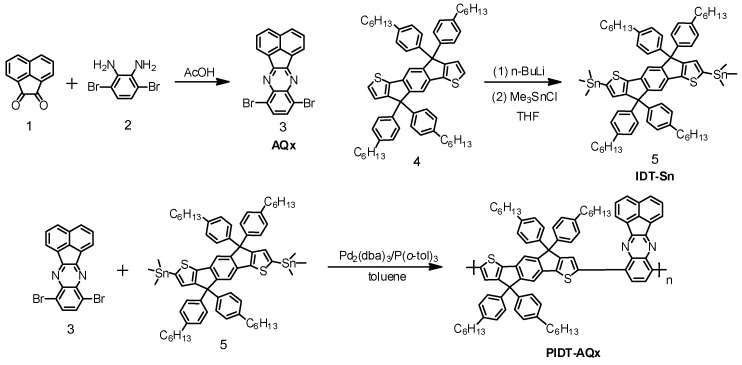
The synthetic routes to monomer AQx, IDT-Sn, and polymer PIDT-AQx.

The synthetic routes of AQx, IDT-Sn, and the polymer PIDT-AQx are shown in Scheme 1. The AQx was prepared through the condensation of acenaphthoquinone (1) and 3,6-dibromobenzene-1,2-diamine (2) by following the literature method with modification [41]. To a mixture of 1 and 2 (molar ratio of 1:1), acetic acid was added and the reaction mixture was stirred at 120 °C for 12 h. After cooling to room temperature, the reaction mixture was poured into water and the crude product was collected by filtration. The crude product was then dried in a vacuum and recrystallized in chloroform to obtain AQx (3) as yellow needle solid with a yield of 80%. The monomer IDT-Sn (5) was synthesized from 4 following our previous report procedure, with a yield of 84% [42]. The polymer PIDT-AQx was synthesized by the Stille polymerization reaction with Pd_2_(dba)_3_/P(*o*-tol)_3_ as catalyst in toluene. PIDT-AQx shows good solubility in most common organic solvents, such as chloroform, toluene, THF, cholorobenzene, and *o*-dichlorobenzene, due to the four hexylbenzene chains in the IDT unit. The number average molecular weight (*M*_n_) and polydispersity index (PDI) was measured to be 79.2 kg/mol and 2.94, respectively, which was performed on a PL-GPC50 instrument (Polytech Instrument, Beijing, China) with THF as eluent. 

### 3.2. Optical and Electrochemical Properties 

**Figure 2:**
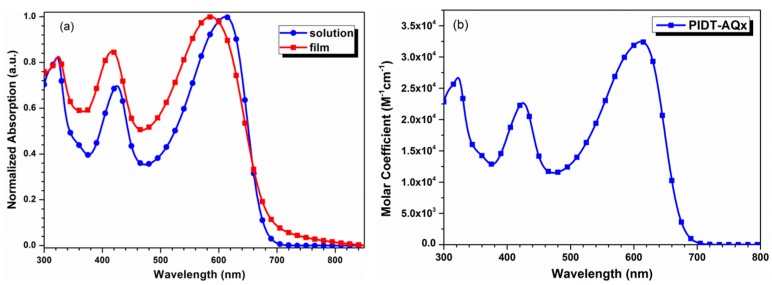
Normalized absorption spectra of PIDT-AQx in chloroform solution and thin film state (**a**) and the molar coefficient of PIDT-AQx in chloroform solution (10^−6^ M) (**b**).

The optical properties of PIDT-AQx in chloroform solution and in the film state were investigated (Figure 2) and the detailed results are collected in Table 1. As shown in Figure 2, the polymer shows similar absorption profiles and has three main absorption peaks in both solution and film states. The absorption peak at ~310 nm was attributed to the n–π* absorption of the building blocks, and the absorption band with a peak at around 420 nm can be assigned to π–π* transitions, whereas the strong low-energy absorption band comes from the strong intramolecular charge transfer (ICT) interaction between the electron-rich IDT and electron-deficient AQx moieties [43]. It is noted that the ICT peak in PIDT-AQx is stronger than that of π–π* transition peak, which is believed to be the narrow electron delocalization in AQx unit due to the naphthalene ring, where other IDT and quinoxaline polymers have a stronger or comparative π–π* peak than that of ICT peak [42,43,44,45]. The absorption maximum in the solution was found to be at 612 nm, however, the absorption maximum of PIDT-AQx in thin film was blue-shifted to 586 nm. Normally, the absorption peak of most conjugated polymers in thin film show a red-shift compared to that in solution. For PIDT-AQx, the blue-shift absorption peak in thin film compared to the solution could be attributed to the twisting of the polymer backbone when the polymer chains packed together, which is a phenomenon also found in some conjugated polymers and small molecules [46,47,48,49]. The absorption edge in solution and thin film states were measured to be 674 and 684 nm, corresponding to an optical band gap (*E*_g_^opt^) of 1.84 and 1.81 eV, respectively. 

**Figure 3:**
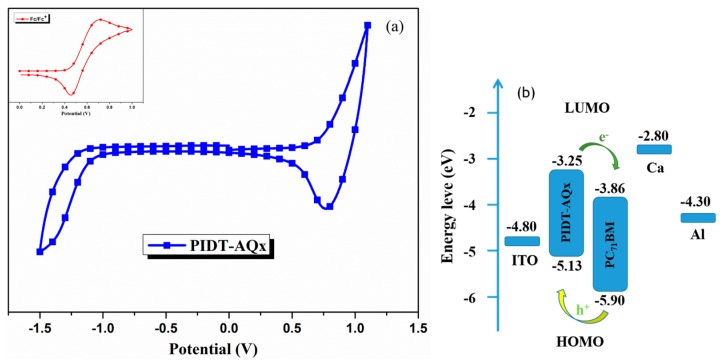
(**a**) The cyclic voltammetry of PIDT-AQx film and (**b**) the schematic energy levels of the PIDT-AQx, PC_71_BM and cathodes (Ca, Al).

To investigate the electrochemical properties of PIDT-AQx, cyclic voltammetry of PIDT-AQx film was determined with ferrocene as the internal standard (Figure 3a). The HOMO and LUMO energy levels were calculated from the oxidation and reduction peaks, respectively (Table 1). As shown in Table 1, the HOMO and LUMO energy levels were found to be −5.13 and −3.25 eV, respectively. The electrochemical band gap (*E*_g_^ec^) was measured to be 1.88 eV, which is very similar with the optical band gap. The difference between the optical and electrochemical band gaps is normally observed in the conjugated polymer, and was mainly induced by the interfacial barrier during the electrochemical measurement. PIDT-AQx exhibited a relatively high HOMO energy level, which could be resulting from the extended conjugation of the polymer backbone. Generally, to facilitate efficient exciton splitting and dissociation, a minimum energy difference of ~0.3 eV between the LUMO energy levels of the donor polymer and the acceptor materials is required [50]. The LUMO energy level of PIDT-AQx was 0.61 eV higher than that of PC_71_BM (−3.86 eV, Figure 3b) [51], therefore an efficient driving force for exciton splitting and charge dissociation could be expected in the PIDT-AQx/PC_71_BM blend. 

**Figure 4:**
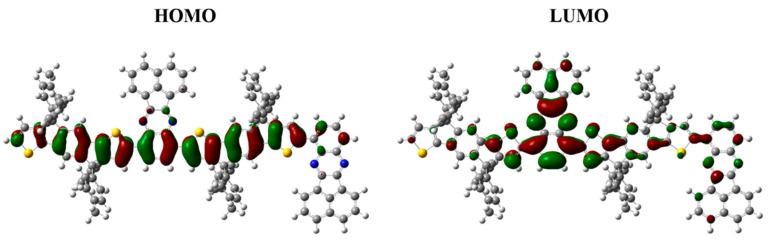
The HOMO and LUMO wave functions of PIDT-AQx.

Figure 4 shows the density functional theory (DFT) theoretical calculation for the distributions of the frontier molecular orbitals of PIDT-AQx. Two repeat units with the methyl side chains replacing the hexyl chains were used to perform the DFT calculation. It is noted that the HOMO and LUMO energy levels were calculated to be −4.49 and −2.29 eV, respectively. A large deviation of DFT calculation from CV measurements was found, since only the dimer with a short methyl chain was used to represent the polymer in the DFT calculation. As shown in Figure 4, the electronic wave function of the HOMO was distributed almost entirely over the conjugated main chain, while the LUMO wave function was mainly localized on the AQx unit.

### 3.3. Photovoltaic Properties

To evaluate the photovoltaic properties of PIDT-AQx, PSC devices with a device architecture of ITO/PEDOT:PSS/PIDT-AQx:PC_71_BM/Ca/Al were fabricated and characterized under 100 mW/cm^2^ (AM 1.5 G) illumination. The PC_71_BM was selected as the acceptor material due to its stronger absorption in visible region over PC_61_BM [52]. The optimized weight ratio of PIDT-AQx:PC_71_BM in the active layer is 1:3. The active layer was spin-coated from the *o*-DCB solution at a total concentration of 40 mg/mL. The detailed device performances for PIDT-AQx:PC_71_BM at different D/A ratios are summarized in Table 2.

**Figure 5:**
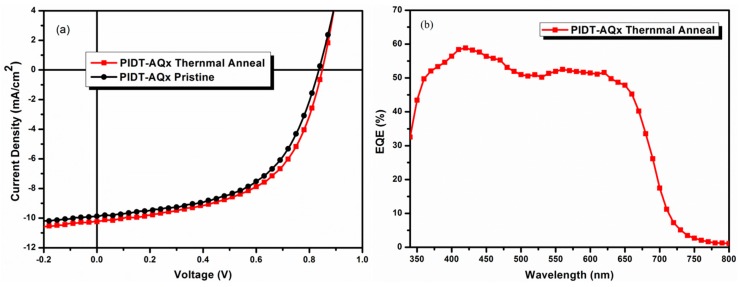
(**a**) The *J*–*V* curves of the pristine device and the annealed device and (**b**) the external quantum efficiency (EQE) spectra of the annealed device.

Figure 5a shows the *J*–*V* curves of the PIDT-AQx/PC_71_BM device under different conditions. It is well known that the pre- or post-treatment of the active layer by either thermal- or solvent-annealing and/or adding the solvent additive is of high importance to enhance the device performance [53,54,55]; however, these methods will raise the difficulty in device engineering and are negative for large-area production. For example, the solvent additives are arduous to control and be removed, and will remain as contaminants because of their high boiling point. The annealing temperature and time are also tedious to be optimized, since the interface defects between the photoactive layer and electrode may be generated when the thermal annealing is performed after the deposition of the electrode [56]. These negative impacts are adverse for the further utilization of ink-jet printing or ultimately roll-to-roll printing techniques. Therefore, it will be much more interesting and important if a promising device performance could be achieved from the pristine blend film, which does not need a solvent additive and the pre- or post-treatments. In our case, we found that the pristine PIDT-AQx/PC_71_BM film-based device achieved a promising PCE of 4.56% with an open-circuit voltage (*V*_oc_) of 0.84 V, a current density (*J*_sc_) of 9.88 mA cm^−2^, and a fill factor (FF) of 55% (Table 2). This value is among the highest PCE reported so far in BHJ PSCs for acenaphthoquinoxaline-based polymer without any thermal or solvent treatments [39]. We also applied a thermal treatment on the PIDT-AQx/PC_71_BM film by annealing at 110 °C for 10 min; it was found that the PCE slightly improved to 4.78% with a similar *V*_oc_ of 0.85 V, an enhanced *J*_sc_ of 10.23 mA cm^−2^, and an identical FF of 55%. This result showed that the post-treatment on the PIDT-AQx/PC_71_BM film has a little effect on the device performance.

To further study the photovoltaic performance of PIDT-AQx under optimal conditions, the external quantum efficiency (EQE) spectra of the PIDT-AQx/PC_71_BM device with thermal annealing was measured. As shown in Figure 5b, the device showed a broad photoresponses from 340 nm to 800 nm. It was found that both PIDT-AQx and PC_71_BM contribute to the EQE values between 340 nm and 500 nm, while the values from 500 nm to 720 nm was mainly contributed by PIDT-AQx. The EQE values are above 50% from 375 nm to 675 nm, with the highest value of 60% at ~425 nm.

**Figure 6:**
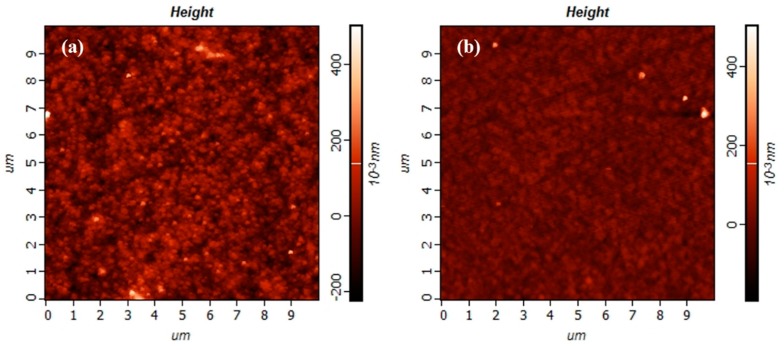
Atomic force microscopy (AFM) height images of PIDT-AQx/PC_71_BM blend film (**a**) as cast and (**b**) thermal annealing at 110 °C for 10 min.

To further understand the difference of photovoltaic performance caused by thermal annealing, the morphology of pristine PIDT-AQx/PC_71_BM thin film and the thermal annealed film was investigated by Atomic force microscopy (AFM) (Figure 6). As shown in Figure 6, the annealed PIDT-AQx/PC_71_BM thin film showed a relatively smooth film, and there was no distinct phase separation for both blend films found. The room-mean-square (RMS) roughness of pristine PIDT-AQx/PC_71_BM blend film was ~1.89 nm, indicating a less favorable surface morphology compared to the thermal annealed PIDT-AQx/PC_71_BM film, which showed a RMS of ~1.51 nm. The results indicate that the phase separation of PIDT-AQx/PC_71_BM blend thin film was more favorable and a more appropriate interpenetrating network may be formed after thermal annealing. This might facilitate the dissociation, transport, and collection of charge carrier, resulting in an improved *J_sc_* and PCE.

## 4. Conclusions

A new wide band gap polymer (PIDT-AQx) based on indacenodithiophene (IDT) and acenaphthoquinoxaline (AQx) was designed and synthesized. The optical band gap of PIDT-AQx was found to be 1.81 eV with a HOMO energy level of −5.13 eV. The polymer solar cells fabricated with the device structure of ITO/PEDOT:PSS/PIDT-AQx:PC_71_BM/Ca/Al showed a promising PCE of 4.56% with a *V*_oc_ of 0.84 V, a *J*_sc_ of 9.88 mA cm^−2^, and a FF of 55% when the active layer was processed directly from the solution without any pre- or post-treatment or additional solvent additives, which simplifies the device fabrication process and is of high importance in the ink-jet printing or ultimately roll-to-roll printing techniques for polymer solar cells. The PCE slightly improved to 4.78% after thermal annealing. The results indicate that the acenaphthoquinoxaline unit is a promising building block in pursing high-performance conjugated polymer solar cells with a simple processing technique.

## Figures and Tables

**Table 1:** Optical and electrochemical properties of PIDT-AQx.

Polymer	λ_max_ (nm)		λ_onset_ (nm)		HOMO (eV) ^a^	LUMO (eV) ^a^	*E*_g_^opt^ (eV)		*E*_g_^ec^ (eV)
	Solution	Film	Solution	Film			Solution	Film	
PIDT-AQx	612	586	674	684	−5.13	−3.25	1.84	1.81	1.88

**^a^** The energy levels were determined in film from cyclic voltammetry (CV) measurement, *E*_g_^ec^ = LUMO – MOMO.

**Table 2:** Photovoltaic performances of the polymer solar cells (PSCs) based on PIDT-AQx:PC_71_BM.

D/A	*V*_oc_ (V)	*J*_sc_ (mA/cm^2^)	FF	PCE_max_ (%)	PCE_avg_^c^ (%)
1:1 ^a^	0.87	5.52	0.37	1.78	1.69 ± 0.09
1:2 ^a^	0.86	9.85	0.52	4.40	4.33 ± 0.07
1:3 ^a^	0.84	9.88	0.55	4.56	4.52 ± 0.04
1:3 ^b^	0.85	10.23	0.55	4.78	4.71 ± 0.07

^a^ Without thermal annealing; ^b^ Thermal annealing at 110 °C for 10 min; ^c^ Average data from 10 cells.

## References

[1] Lu L., Zheng T., Wu Q., Schneider A.M., Zhao D., Yu L. (2015). Recent advances in bulk heterojunction polymer solar cells. Chem. Rev..

[2] Yao H., Ye L., Zhang H., Li S., Zhang S., Hou J. (2016). Molecular design of benzodithiophene-based organic photovoltaic materials. Chem. Rev..

[3] Ma Y., Kang Z., Zheng Q. (2017). Recent advances in wide bandgap semiconducting polymers for polymer solar cells. J. Mater. Chem. A.

[4] Gao Y., Liu M., Zhang Y., Liu Z., Yang Y., Zhao L. (2017). Recent development on narrow bandgap conjugated polymers for polymer solar cells. Polymers.

[5] Liu C., Wang K., Gong X., Heeger A.J. (2016). Low bandgap semiconducting polymers for polymeric photovoltaics. Chem. Soc. Rev..

[6] Jung J.W., Jo J.W., Jung E.H., Jo W.H. (2016). Recent progress in high efficiency polymer solar cells by rational design and energy level tuning of low bandgap copolymers with various electron-withdrawing units. Org. Electron..

[7] Hu S., Bao X., Liu Z., Wang T., Du Z., Wen S., Wang N., Han L., Yang R. (2014). Benzothiadiazole[1,2-b:4,3-b′]dithiophene, a new ladder-type multifused block: Synthesis and photovoltaic application. Org. Electron..

[8] Hu Z., Li X.-D., Zhang W., Liang A., Ye D., Liu Z., Liu J., Liu Y., Fang J. (2014). Synthesis and photovoltaic properties of solution-processable star-shaped small molecules with triphenylamine as the core and alkyl cyanoacetate or 3-ethylrhodanine as the end-group. RSC Adv..

[9] Liu Z., Wu Y., Zhang Q., Gao X. (2016). Non-fullerene small molecule acceptors based on perylene diimides. J. Mater. Chem. A.

[10] Dou L., Liu Y., Hong Z., Li G., Yang Y. (2015). Low-bandgap near-IR conjugated polymers/molecules for organic electronics. Chem. Rev..

[11] Liu Y., Liu J., Zhang L., Fang J., Zhang W., Liu Z. (2014). Non-fullerene organic small molecule electron-acceptors. Chin. J. Org. Chem..

[12] Marrocchi A., Facchetti A., Lanari D., Santoro S., Vaccaro L. (2016). Click-chemistry approaches to π-conjugated polymers for organic electronics applications. Chem. Sci..

[13] Yu G., Gao J., Hummelen J.C., Wudl F., Heeger A.J. (1995). Polymer photovoltaic cells: Enhanced efficiencies via a network of internal donor-acceptor heterojunctions. Science.

[14] Heeger A.J. (2014). 25th anniversary article: Bulk heterojunction solar cells: Understanding the mechanism of operation. Adv. Mater..

[15] Facchetti A. (2013). Polymer donor–polymer acceptor (all-polymer) solar cells. Mater. Today.

[16] Kang H., Lee W., Oh J., Kim T., Lee C., Kim B.J. (2016). From fullerene–polymer to all-polymer solar cells: The importance of molecular packing, orientation, and morphology control. Acc. Chem. Res..

[17] Wang K., Liu C., Meng T., Yi C., Gong X. (2016). Inverted organic photovoltaic cells. Chem. Soc. Rev..

[18] Wang F., Tan Z.-A., Li Y. (2015). Solution-processable metal oxides/chelates as electrode buffer layers for efficient and stable polymer solar cells. Energy Environ. Sci..

[19] Liao H.-C., Ho C.-C., Chang C.-Y., Jao M.-H., Darling S.B., Su W.-F. (2013). Additives for morphology control in high-efficiency organic solar cells. Mater. Today.

[20] Zhang L., Ma W. (2017). Morphology optimization in ternary organic solar cells. Chin. J. Polym. Sci..

[21] Leclerc N., Chávez P., Ibraikulov O., Heiser T., Lévêque P. (2016). Impact of backbone fluorination on π-conjugated polymers in organic photovoltaic devices: A review. Polymers.

[22] Zeng H., Zhu X., Liang Y., Guo X. (2015). Interfacial layer engineering for performance enhancement in polymer solar cells. Polymers.

[23] Zhou H., Yang L., You W. (2012). Rational design of high performance conjugated polymers for organic solar cells. Macromolecules.

[24] Chen S., Liu Y., Zhang L., Chow P.C.Y., Wang Z., Zhang G., Ma W., Yan H. (2017). A wide-bandgap donor polymer for highly efficient non-fullerene organic solar cells with a small voltage loss. J. Am. Chem. Soc..

[25] Lin Y., Zhao F., Wu Y., Chen K., Xia Y., Li G., Prasad S.K.K., Zhu J., Huo L., Bin H. (2017). Mapping polymer donors toward high-efficiency fullerene free organic solar cells. Adv. Mater..

[26] Fan B., Zhang K., Jiang X.-F., Ying L., Huang F., Cao Y. (2017). High-performance nonfullerene polymer solar cells based on imide-functionalized wide-bandgap polymers. Adv. Mater..

[27] Guo B., Li W., Guo X., Meng X., Ma W., Zhang M., Li Y. (2017). High efficiency nonfullerene polymer solar cells with thick active layer and large area. Adv. Mater..

[28] Xue L., Yang Y., Xu J., Zhang C., Bin H., Zhang Z.-G., Qiu B., Li X., Sun C., Gao L. (2017). Side chain engineering on medium bandgap copolymers to suppress triplet formation for high-efficiency polymer solar cells. Adv. Mater..

[29] Zhou C., Zhang G., Zhong C., Jia X., Luo P., Xu R., Gao K., Jiang X., Liu F., Russell T.P. (2017). Toward high efficiency polymer solar cells: Influence of local chemical environment and morphology. Adv. Energy Mater..

[30] Mai J., Lau T.-K., Li J., Peng S.-H., Hsu C.-S., Jeng U.S., Zeng J., Zhao N., Xiao X., Lu X. (2016). Understanding morphology compatibility for high-performance ternary organic solar cells. Chem. Mater..

[31] Ye L., Xiong Y., Li S., Ghasemi M., Balar N., Turner J., Gadisa A., Hou J., O’Connor B.T., Ade H. (2017). Precise manipulation of multilength scale morphology and its influence on eco-friendly printed all-polymer solar cells. Adv. Funct. Mater..

[32] Zhao J., Zhao S., Xu Z., Qiao B., Huang D., Zhao L., Li Y., Zhu Y., Wang P. (2016). Revealing the effect of additives with different solubility on the morphology and the donor crystalline structures of organic solar cells. ACS Appl. Mater. Interfaces.

[33] Zhao W., Li S., Yao H., Zhang S., Zhang Y., Yang B., Hou J. (2017). Molecular optimization enables over 13% efficiency in organic solar cells. J. Am. Chem. Soc..

[34] Cui Y., Yao H., Gao B., Qin Y., Zhang S., Yang B., He C., Xu B., Hou J. (2017). Fine-tuned photoactive and interconnection layers for achieving over 13% efficiency in a fullerene-free tandem organic solar cell. J. Am. Chem. Soc..

[35] Li Y., Gu M., Pan Z., Zhang B., Yang X., Gu J., Chen Y. (2017). Indacenodithiophene: A promising building block for high performance polymer solar cells. J. Mater. Chem. A.

[36] Liang C., Wang H. (2017). Indacenodithiophene-based D-A conjugated polymers for application in polymer solar cells. Org. Electron..

[37] Ma Y., Chen S.-C., Wang Z., Ma W., Wang J., Yin Z., Tang C., Cai D., Zheng Q. (2017). Indacenodithiophene-based wide bandgap copolymers for high performance single-junction and tandem polymer solar cells. Nano Energy.

[38] Zhang Z., Peng Q., Yang D., Chen Y., Huang Y., Pu X., Lu Z., Jiang Q., Liu Y. (2013). Novel conjugated polymers with planar backbone bearing acenaphtho[1,2-b]quinoxaline acceptor subunit for polymer solar cells. Synth. Met..

[39] Fang T., Lu Z., Lu H., Li C., Li G., Kang C., Bo Z. (2015). The enhanced photovoltaic performance of fluorinated acenaphtho[1,2-b]quinoxaline based low band gap polymer. Polymer.

[40] Zou Y., Guan Z., Zhang Z., Huang Y., Wang N., Lu Z., Jiang Q., Yu J., Liu Y., Pu X. (2012). Synthesis and photovoltaic properties of conjugated copolymers bearing planar acenaphtho[1,2-b]quinoxaline moiety with deep homo level. J. Mater. Sci..

[41] Guo Z.-H., Lei T., Jin Z.-X., Wang J.-Y., Pei J. (2013). T-shaped donor—Acceptor molecules for low-loss red-emission optical waveguide. Org. Lett..

[42] Zhang Y., Zou J., Yip H.-L., Chen K.-S., Zeigler D.F., Sun Y., Jen A.K.Y. (2011). Indacenodithiophene and quinoxaline-based conjugated polymers for highly efficient polymer solar cells. Chem. Mater..

[43] Zhang Y., Zou J., Yip H.-L., Chen K.-S., Davies J.A., Sun Y., Jen A.K.Y. (2011). Synthesis, characterization, charge transport, and photovoltaic properties of dithienobenzoquinoxaline- and dithienobenzopyridopyrazine-based conjugated polymers. Macromolecules.

[44] Sulas D.B., Yao K., Intemann J.J., Williams S.T., Li C.-Z., Chueh C.-C., Richards J.J., Xi Y., Pozzo L.D., Schlenker C.W. (2015). Open-circuit voltage losses in selenium-substituted organic photovoltaic devices from increased density of charge-transfer states. Chem. Mater..

[45] He R., Yu L., Cai P., Peng F., Xu J., Ying L., Chen J., Yang W., Cao Y. (2014). Narrow-band-gap conjugated polymers based on 2,7-dioctyl-substituted dibenzo[*a,c*]phenazine derivatives for polymer solar cells. Macromolecules.

[46] Chochos C.L., Katsouras A., Gasparini N., Koulogiannis C., Ameri T., Brabec C.J., Avgeropoulos A. (2017). Rational design of high-performance wide-bandgap (≈2 eV) polymer semiconductors as electron donors in organic photovoltaics exhibiting high open circuit voltages (≈1 V). Macromol. Rapid Commun..

[47] Xu Y.-X., Chueh C.-C., Yip H.-L., Chang C.-Y., Liang P.-W., Intemann J.J., Chen W.-C., Jen A.K.Y. (2013). Indacenodithieno[3,2-*b*]thiophene-based broad bandgap polymers for high efficiency polymer solar cells. Polym. Chem..

[48] Silvestri F., Marrocchi A., Seri M., Kim C., Marks T.J., Facchetti A., Taticchi A. (2010). Solution-processable low-molecular weight extended arylacetylenes: Versatile *p*-type semiconductors for field-effect transistors and bulk heterojunction solar cells. J. Am. Chem. Soc..

[49] Marrocchi A., Silvestri F., Seri M., Facchetti A., Taticchi A., Marks T.J. (2009). Conjugated anthracene derivatives as donor materials for bulk heterojunction solar cells: Olefinic versus acetylenic spacers. Chem. Commun..

[50] Scharber M.C., Mühlbacher D., Koppe M., Denk P., Waldauf C., Heeger A.J., Brabec C.J. (2006). Design rules for donors in bulk-heterojunction solar cells—Towards 10% energy-conversion efficiency. Adv. Mater..

[51] Zhang L., Yang T., Shen L., Fang Y., Dang L., Zhou N., Guo X., Hong Z., Yang Y., Wu H. (2015). Toward highly sensitive polymer photodetectors by molecular engineering. Adv. Mater..

[52] Wienk M.M., Kroon J.M., Verhees W.J.H., Knol J., Hummelen J.C., van Hal P.A., Janssen R.A.J. (2003). Efficient methano[70]fullerene/MDMO-PPV bulk heterojunction photovoltaic cells. Angew. Chem..

[53] Dang D., Wang X., Zhi Y., Meng L., Bao X., Yang R., Zhu W. (2016). Synthesis and characterization of D-A-A type regular terpolymers with narrowed band-gap and their application in high performance polymer solar cells. Org. Electron..

[54] Li Z., Xu X., Zhang W., Meng X., Ma W., Yartsev A., Inganäs O., Andersson M.R., Janssen R.A.J., Wang E. (2016). High performance all-polymer solar cells by synergistic effects of fine-tuned crystallinity and solvent annealing. J. Am. Chem. Soc..

[55] Wan Q., Guo X., Wang Z., Li W., Guo B., Ma W., Zhang M., Li Y. (2016). 10.8% efficiency polymer solar cells based on PTB7-Th and PC71BM via binary solvent additives treatment. Adv. Funct. Mater..

[56] Yang X., Loos J. (2007). Toward high-performance polymer solar cells: The importance of morphology control. Macromolecules.

